# More Studies are Needed on the Link between Metformin and Decreased Mortality in Diabetic COVID-19 Patients

**DOI:** 10.4269/ajtmh.20-0766

**Published:** 2020-07-24

**Authors:** Marinos Fysekidis, Régis Cohen, Abdallah Al-Salameh

**Affiliations:** Department of Endocrinology; Hôpital Privé de I’Est Parisien; Aulnay-sous-Bois, France; E-mail: fisekidis_marinos@hotmail.com; Department of Internal Medicine; Centre Hospitalier de Saint-Denis; Saint-Denis, France; E-mail: regis.cohen@ch-stenis.fr; Department of Endocrinology; Diabetes Mellitus and Nutrition; Amiens University Hospital; Amiens, France; E-mail: abdallahalsalameh@hotmail.com

Dear Sir,

We would like to congratulate Luo et al.^1^ for their study about the association between metformin and decreased mortality in patients with COVID-19 and diabetes. Their findings are very interesting. However, the study by Luo et al.^1^ raises a number of issues that should be addressed.

More than 60 years after its introduction for the treatment of type 2 diabetes mellitus, metformin remains among the most widely prescribed drugs worldwide. Beyond its antidiabetic, cardiovascular, and reno-protective actions, metformin has pleiotropic effects, including cell protection, cancer prevention, immunomodulatory properties, anti-inflammatory effects, and adjuvant antimicrobial benefits in multiple infectious diseases. Interestingly, metformin has been used to treat influenza.^2^ However, metformin has some adverse effects and contraindications.^3^ It should be stopped in patients with concomitant kidney and liver failure because kidney failure leads to metformin accumulation and liver failure reduces lactate elimination, increasing the risk of lactic acidosis.^4^ Therefore, diabetic patients who do not take metformin are likely those with organ failure (heart failure, chronic obstructive pulmonary disease, chronic renal failure, or cirrhosis) precluding its administration. These patients are also at high risk of developing severe and critical COVID-19 characterized by respiratory and multi-organ failure, mechanical ventilation, and death. Current guidelines also recommend withdrawing metformin during periods of suspected tissue hypoxia.^5^

We note a number of concerns regarding the study by Luo et al.^1^ First, there was no definition for diabetes. In the context of COVID-19, newly diagnosed diabetes is characterized by high mortality, and these patients will not be taking metformin on admission.^6^ Moreover, no data were available about diabetes duration and complications. Long-standing diabetes is prone to microvascular and macrovascular complications which are associated with mortality in diabetic patients with COVID-19.^7^

Second, although the authors reported about many coexisting diseases and treatments, some important data were missed, such as the prevalence of obesity and treatment with angiotensin-converting enzyme inhibitors and angiotensin receptor blockers. A growing body of evidence suggests that morbid obesity is associated with a severe clinical course in COVID-19.^8,9^

Third, the study groups were not well balanced regarding the prevalence of comorbidities and antiviral treatments. The authors listed comorbidities separately, but, if we compare the groups, the metformin group had significantly fewer underlying conditions (75/104, 72%) than the non-metformin group (149/179, 83%; *P* = 0.026). Moreover, for every antiviral treatment listed in Table 3 of the article, the proportion of patients treated with antiviral treatment was higher in the metformin group than in the non-metformin group.

Last, we did not understand the results of the multivariable analysis. There was an odds ratio (OR) of 0.23 for the association between metformin and in-hospital mortality based on “univariable analysis” (Table 4), whereas in Table 3, the OR for metformin was 4.36. On the other hand, the authors did not have enough events (25 deaths), to create an eight-variable model.

As such, Luo’s conclusions must be taken with caution. The presence of recruitment bias, confounders, and inadequate statistical power can impact the validity of statistical analysis. As noted by the authors, a randomized prospective study is needed to better test the value of treatment with metformin for COVID-19.
